# Development of an oxygenation culture method for activating the liver-specific functions of HepG2 cells utilizing a collagen vitrigel membrane chamber

**DOI:** 10.1007/s10616-015-9934-1

**Published:** 2015-12-09

**Authors:** Ayumi Oshikata-Miyazaki, Toshiaki Takezawa

**Affiliations:** Division of Animal Sciences, National Institute of Agrobiological Sciences, Ohwashi 1-2, Tsukuba, Ibaraki 305-8634 Japan

**Keywords:** ADME, Collagen vitrigel, CYP3A4, HepG2 cells, Liver, Oxygenation

## Abstract

We recently developed a collagen vitrigel membrane (CVM) chamber possessing a scaffold composed of high-density collagen fibrils. In this study, we first confirmed that the advantage of CVM chamber in comparison to the traditional culture chamber with porous polyethylene terephthalate membrane is to preserve a culture medium poured in its inside even though the under side is not a liquid phase but solid and gas phases. Subsequently, we designed three different culture systems to grow HepG2 cells in a culture medium (liquid phase) on the CVM which the under side is a culture medium, a plastic surface (solid phase) or 5 % CO_2_ in air (gas phase) and aimed to develop a brief culture method useful for activating the liver-specific functions and analyzing the pharmacokinetics of fluorescein diacetate. HepG2 cells cultured for 2 days on the liquid–solid interface and subsequently for 1 day on the liquid–gas interface represented excellent cell viability and morphology in comparison to the others, and remarkably improved albumin secretion and urea synthesis to almost the same level of freshly isolated human hepatocytes and CYP3A4 activity to about half the level of differentiated HepaRG cells. Also, the cells rapidly absorbed fluorescein diacetate, distributed it in cytosol, metabolized it into fluorescein, and speedily excreted fluorescein into both bile canaliculus-like networks and extracellular solution. These data suggest that hepatic structure and functions of monolayered HepG2 cells can be induced within a day after the oxygenation from beneath the CVM.

## Introduction

It was previously reported that HepG2 cells, a human hepatoma cell line, inherently possess most biochemical and histological features of normal human parenchymal hepatocytes (Bouma et al. 1989; Knowles et al. 1980). Therefore, HepG2 cells that can sufficiently express liver-specific functions are considered as a valuable candidate of human hepatic cell source for drug development. The microenvironment of parenchymal hepatocytes expressing liver-specific functions in vivo is composed of extracellular matrix, extracellular fluid containing the appropriate amount of dissolved oxygen, and non-parenchymal cells. Therefore, the microenvironment of HepG2 cells in the culture system in vitro has been devised for activating their liver-specific functions. For example, a collagen gel substratum modulates liver transcription factors in HepG2 cells (Dipersio et al. 1991). A recombinant fibronectin protein-immobilized plate induced albumin expression in HepG2 cells (Nishida and Taniguchi 2013). Also, the continuous oxygenation culture system utilizing a gas-permeable PDMS (polydimethylsiloxane) was used for fabricating both thick multilayers and large multicellular spheroids with excellent viability, resulting in the improvement of albumin secretion of HepG2 cells (Anada et al. 2012; Evenou et al. 2010). Further, the co-culture with endothelial cells or fibroblasts promoted the expression of cytochrome P450 (CYP) enzymes such as CYP1A2 and CYP3A4 in HepG2 cells (Kikuchi et al. 2009; Ohono et al. 2008). However, the above culture systems are still insufficient for activating various liver-specific functions of HepG2 cells equivalent to human primary parenchymal hepatocytes in spite of the fact that they require not only complicate manipulations but also a long period of more than 10 days.

Meanwhile, a collagen vitrigel membrane (CVM), we have developed, is composed of high-density collagen fibrils equivalent to connective tissues in vivo and is easily handled with tweezers. It possesses excellent strength, transparency and permeability of protein with high molecular weight. Also, it functions as an excellent scaffold for fabricating three-dimensional cell culture models, as cells can be cultured on both sides. The advantage of protein-permeable CVM scaffold is to facilitate the fabrication of crosstalk models between two different cell types useful for paracrine assays in vitro (Takezawa et al. 2004, 2007a, b). Recently, we developed a mass fabrication technology of not only a thin but also thick CVM in a dried state, i.e. a collagen xerogel membrane (CXM) with an excellent handling ability. The CXM was pasted onto the bottom-side edge of a plastic cylinder with an inner-outer diameter of 11–13 mm and a length of 15 mm, and a couple of hangers suitable for setting it in a well of a 12-well plate was connected onto the top-side edge of it, resulting in the fabrication of a CXM chamber that can easily convert into a CVM chamber by rehydration (Takezawa et al. 2012). Eye irritancy of chemicals was successfully predicted by analyzing time-dependent profiles of TEER (transepithelial electrical resistance) values for 3 min after exposing chemicals to a human corneal epithelium model fabricated in the CVM chamber, so that we named it “Vitrigel-EIT (eye irritancy test) method” (Takezawa et al. 2011; Yamaguchi et al. 2013). Also, we developed the CXM chamber containing two compartments useful for fabricating crosstalk models between two different cell types that another plastic cylinder was connected to the CXM chamber containing one compartment by taping the edges of the two cylinders together via the CXM (Aoki et al. 2014). The advantage of the CVM chambers in comparison to the traditional culture chambers with porous polyethylene terephthalate (PET) membrane can easily provide various culture models of not only single surface but also double surface and also their cryo-sections useful for immune-histology. Meanwhile, the disadvantage of the CVM chambers is that its membrane is swelled and consequently slightly deflected when the CXM chamber is dehydrated.

**Fig. 1:**
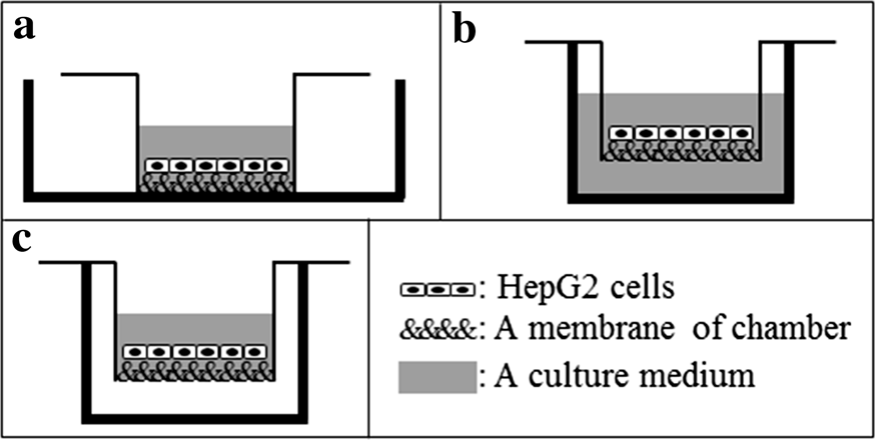
Schematic illustration of three different culture methods of HepG2 cells using culture chambers. “Liquid–solid interface” culture method (**a**), “liquid–liquid interface” culture method (**b**), and “liquid–gas interface” culture method (**c**). *Thin black line* with the sequence of “&”-symbols, *gray zone* and *white zone* represent a culture chamber, a culture medium and a humidified gas of 5 % CO_2_ in air, respectively. Here, the sequence of “&”-symbols represents a membrane of culture chambers; CVM of CVM chambers or a porous PET membrane of control Millicell chambers. Also, *thick black line* in (**a**) represents a plastic culture dish with a diameter of 60 mm and that in (**b**) and (**c**) does a well of 12 well-plastic culture plate

To establish a unique and brief culture system that can activate the hepatic functions of HepG2 cells, we propose a novel culture method with continuous oxygenation using a CVM chamber excellent in not only handling itself but also fabricating various co-culture models. As the first step, we aimed to develop a novel culture method with continuous oxygenation towards HepG2 cells using a CVM chamber. In this study, we investigated liver-specific functions such as albumin secretion, urea synthesis and cellular CYP3A4 activity levels and analyzed the absorption, distribution, metabolism, and excretion (ADME) behavior of fluorescein diacetate (FD) as a model chemical in three unique culture methods for growing HepG2 cells in the CVM chamber as follows. First is an equivalent to an original method using a CVM adhered onto the surface of usual culture dish as a culture substratum, and that is a liquid–solid interface culture method in which the down side of CVM is a solid of plastic surface. Second is an orthodox method using a CVM chamber set in a well of 12-well plate, and that is a liquid–liquid interface culture method in which the down side of CVM is a liquid of culture medium. Third is a novel method expecting continuous oxygenation via a CVM, and that is a liquid–gas interface culture method in which the down side of CVM is a gas of 5 % CO_2_ in air (Fig. 1).

## Materials and methods

### Materials

Materials used in the present study were obtained as follows: Dulbecco’s modified Eagle medium (DMEM), *N*-2-hydroxyethylpiperazine-*N*′-2-ethanesulfonic acid (HEPES), antibiotics (penicillin and streptomycin), and LIVE/DEAD Viability/Cytotoxicity kit were from Life Technologies Corp. (Grand Island, NY, USA); fetal bovine serum (FBS), Hanks’ balanced salt solution (HBSS), dimethyl sulfoxide (DMSO) and fluorescein diacetate (FD) from Sigma-Aldrich (St. Louis, MO, USA); Glucose C-II Test Wako and Urea nitrogen-B Test Wako from Wako Pure Chemical Industries (Osaka, Japan); Human Albumin ELISA Quantitation Set from Bethyl Laboratories (Montgomery, TX, USA); P450-Glo CYP3A4 Assay with Luciferin-IPA from Promega (Madison, WI, USA); Millicell from Merck Millipore (Darmstadt, Germany); all other materials and chemicals not specified above were of the highest grade.

### Culture of HepG2 cells

A human hepatoma cell line derived from hepatocellular carcinoma (HepG2 cells: RCB no. 1648) was obtained from RIKEN BioResource Center (Tsukuba, Japan). HepG2 cells were cultured in DMEM supplemented with 10 % heat-inactivated FBS, 20 mM HEPES, 100 units/ml penicillin and 100 µg/ml streptomycin at 37 °C in a humidified atmosphere of 5 % CO_2_ and 95 % air.

### Compatibility tests of CVM chambers for designing three culture methods

CVM chambers and their control Millicell chambers with a 1.0 μm-porous PET membrane or a 0.4 μm-porous PET membrane for designing three culture methods were subjected to the compatibility tests to confirm their capacity of preserving a culture medium and the transparency important for observing cell morphology by a phase-contrast microscope. To design the solid plastic surface for the down side of each chamber, 0.50 ml of phosphate buffered saline (PBS) was poured into a CVM chamber and its control Millicell chambers and subsequently each chamber was set on the surface of a culture dish with a diameter of 60 mm pre-poured with 2.0 ml of PBS and kept for 10 min to completely rehydrate each membrane. Then, the inside and outside PBS of each chamber was removed and each membrane was dried out well in a clean bench at room temperature for 2 h. To design the liquid culture medium for the down side of each chamber, each chamber was set in a well of 12-well plate pre-poured with 2.0 ml of culture medium. To design the gas for the down side of each chamber, each chamber was set in a well of 12-well plate in air. The compatibility test of these chambers for designing liquid–solid, liquid–liquid and liquid–gas interface culture methods was performed as follows. 0.50 ml of culture medium was poured in the inside of each chamber pre-set on the solid plastic, liquid culture medium and gas surfaces. Then, the capability for preserving the inside culture medium was examined by keeping each condition for more than 2 h. Also, the transparency of each membrane was evaluated by phase-contrast microscopic observation.

### Three culture methods for growing HepG2 cells

Immediately before seeding cells, the CVM of each CVM chamber was rehydrated by pouring 0.5 ml of a culture medium, keeping it for 10 min and removing it. HepG2 cells suspended in 0.5 ml of culture medium were poured in the CVM chambers adhered on the solid plastic surface of a culture dish with a diameter of 60 mm at the initial seeding cell density of 5.0 × 10^4^ cells/cm^2^ and cultured for 48 h to induce the uniform cell growth on the liquid–solid interface. Subsequently, three different culture methods on the liquid–solid, liquid–liquid or liquid–gas interface were performed as follows. In the liquid–solid interface culture method the prior culture was merely maintained for additional 24 h. In the liquid–liquid and liquid–gas interface culture methods each CVM chamber in which cells uniformly proliferated was transferred to a well of 12-well plate in which 2.0 ml of culture medium was pre-poured and an empty well of it, respectively. Then, the cells in each CVM chamber were cultured for additional 24 h. In this transfer process, each CVM chamber was gently detached from the solid plastic surface by pouring 2.0 ml of culture medium in the outside of the chambers. Meanwhile, in the control culture method the control Millicell chambers with a 1.0 μm-porous PET membrane were pre-set in the wells of a 12-well plate. Then, HepG2 cells suspended in 0.5 ml of culture medium were poured in the control Millicell chambers at initial seeding cell density of 5.0 × 10^4^ cells/cm^2^ and 2.0 ml of culture medium was poured in each well, and subsequently the cells were cultured for 72 h. For all culture methods, the medium was changed after 24 and 48 h. Media were collected from the 1-day period between the 48–72 h time points and analyzed using biochemical assays for glucose consumption, albumin secretion and urea synthesis. Also, the cell morphology was periodically observed with a phase-contrast microscope.

### Cell viability assay

Cell viability was analyzed by using LIVE/DEAD Viability/Cytotoxicity Kit composed of calcein-AM and ethidium homodimer-1. The cells cultured in each culture method at 72 h were washed twice with 0.5 ml of PBS. Then, the cells were incubated for 10 min in 0.5 ml of HBSS containing 4 µM calcein-AM and 2 µM ethidium homodimer-1 at 37 °C. Subsequently, all chamber formats except for the CVM chambers in the liquid–solid interface culture method were transferred onto a solid plastic surface to obtain optimal focus under the microscope. Then, the cells in each chamber were observed with a fluorescent microscope.

### Biochemical assay

Glucose consumption, albumin secretion and urea synthesis levels in the conditioned medium accumulated for 24 h from 48 to 72 h in each culture method were measured in accordance with each manufacture’s protocol for the Glucose C-II Test Wako, Human Albumin ELISA Quantitation Set and Urea nitrogen-B Test Wako, respectively. Then, the cell number in each culture chamber at 72 h was estimated from the glucose consumption level (Kiyota et al. 2007) and the relative values of albumin secretion and urea synthesis levels per 1.0 × 10^6^ cells were calculated. Also, the cellular CYP3A4 activity at 72 h in each culture method was basically measured using a GloMax luminometer from Promega (Madison, WI, USA) in accordance with the manufacture’s protocol for P450-Glo CYP3A4 assay with Luciferin-IPA except for adapting the amount of reagents corresponding to the culture area.

### ADME analysis using FD

The cells cultured in each culture method at 72 h were subjected to the ADME analysis using FD basically in accordance with a previous report (Oshima et al. 2008). Each culture medium in the inside of chambers was changed to 0.5 ml of a fresh medium containing 250 µg/ml FD and subsequently the cells in each culture method were cultured at 37 °C for 60 min to sufficiently incorporate FD and metabolize the distributed FD into fluorescein in cytosol of each cell. Then, the culture medium was removed and the cells were rinsed two times with 0.5 ml of HBSS to discontinue the FD incorporation. Subsequently, all chamber formats except for the CVM chambers in the liquid–solid interface culture method were transferred onto a solid plastic surface to obtain optimal focus under the microscope. Then, the cells in each chamber were incubated for additional 60 min in 0.5 ml of HBSS at room temperature and the excretion behavior of metabolized fluorescein was observed at 10 and 60 min with a fluorescent microscope.

### Statistical analysis

Data obtained from three independent experiments were analyzed by the Student’s *t* test. Values are presented as the mean ± standard deviations. Values of *P < 0.01 and **P < 0.001 were considered to indicate statistically significant differences.

## Results

### Advantages of CVM chambers for designing three culture methods

**Fig. 2:**
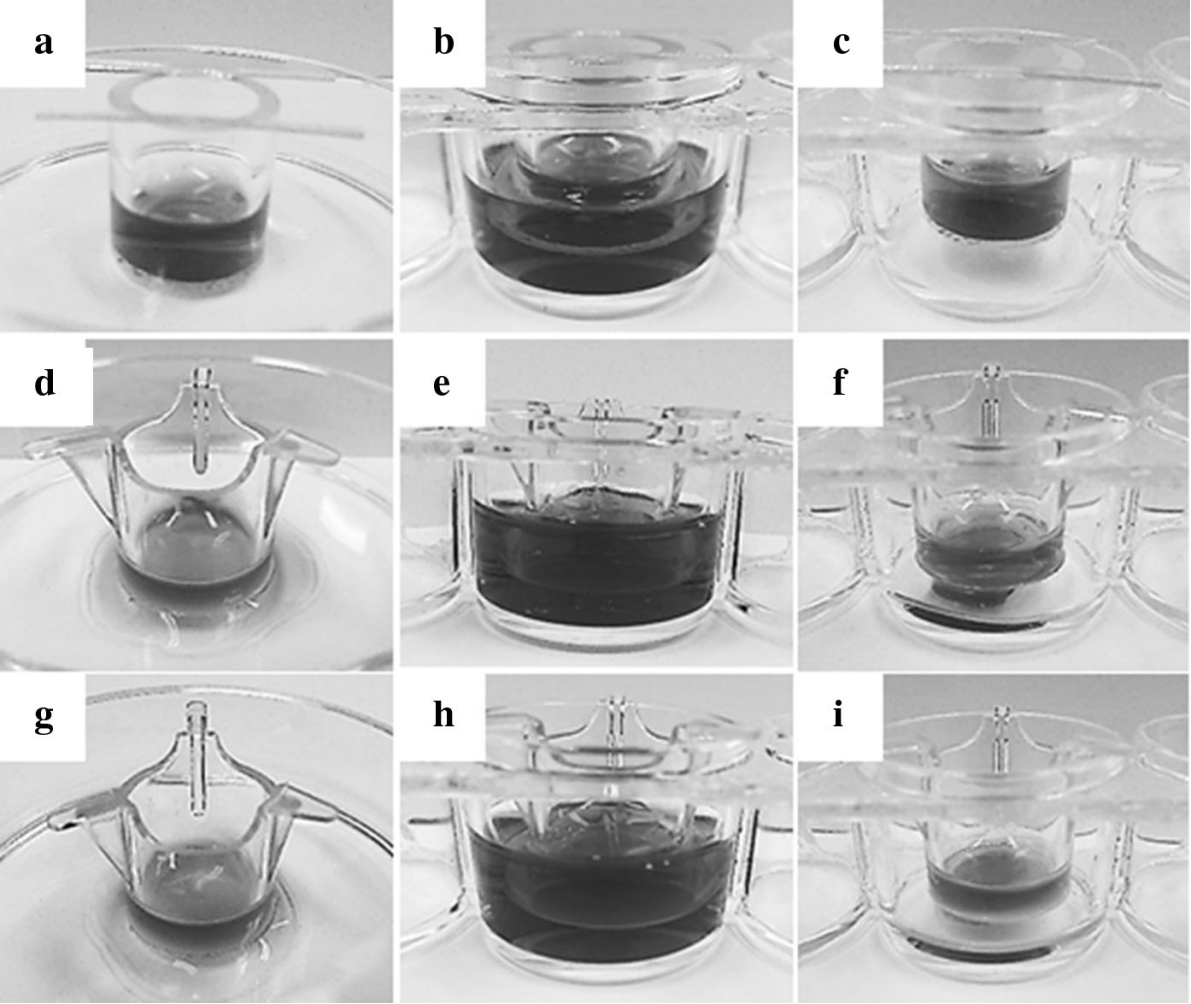
Gross observation showing advantages of CVM chambers in comparison to control Millicell chambers. In order to design three different culture methods described in Fig. 1 using CVM chambers and their control Millicell chambers, 0.50 ml of culture medium was poured in the inside of each chamber pre-set on the solid (**a**, **d**, **g**), liquid (**b**, **e**, **h**), and gas (**c**, **f**, **i**) surfaces and the capability for preserving the inside culture medium was examined by keeping each condition for 2 h. The chambers in (**a**–**c**), (**d**–**f**), and (**g**–**i**) represent CVM chambers, control Millicell chambers with a 1.0 µm-porous PET membrane, and those with a 0.4 µm-porous PET membrane, respectively

The compatibility tests for designing three culture methods (Fig. 1) were performed using CVM chambers and their control Millicell chambers with a 1.0 µm- or a 0.4 µm-porous PET membrane pre-set on solid plastic, liquid culture medium and gas surfaces. CVM chambers pre-set on all conditions and the control Millicell chambers pre-set on the liquid culture medium surface preserved the culture medium for 2 h (Fig. 2a–c, e, h) and this phenomena continued more than 3 days (data not shown). However, the control Millicell chambers pre-set on the solid plastic and gas surfaces did not preserve the culture medium and about half volume of it was leaked within 2 h with no relation to the pore size of PET membrane (Fig. 2d, f, g, i). These data demonstrate that the Millicell chambers with porous PET membrane pre-set on the liquid culture surface can be merely available for designing the control culture method.

Also, the phase-contrast microscopic observations revealed that the CVMs of CVM chambers pre-set on all conditions were sufficiently transparent although the 1.0 µm- and 0.4 µm-porous PET membranes of the Millicell chambers pre-set on the liquid culture surface were slightly and heavily opaque due to the pores, respectively (data not shown). These data suggest that the Millicell chambers with a 1.0 µm-porous PET membrane in comparison to those with a 0.4 µm-porous PET membrane are appropriate for designing the control culture method.

### Growth and viability of HepG2 cells

**Fig. 3:**
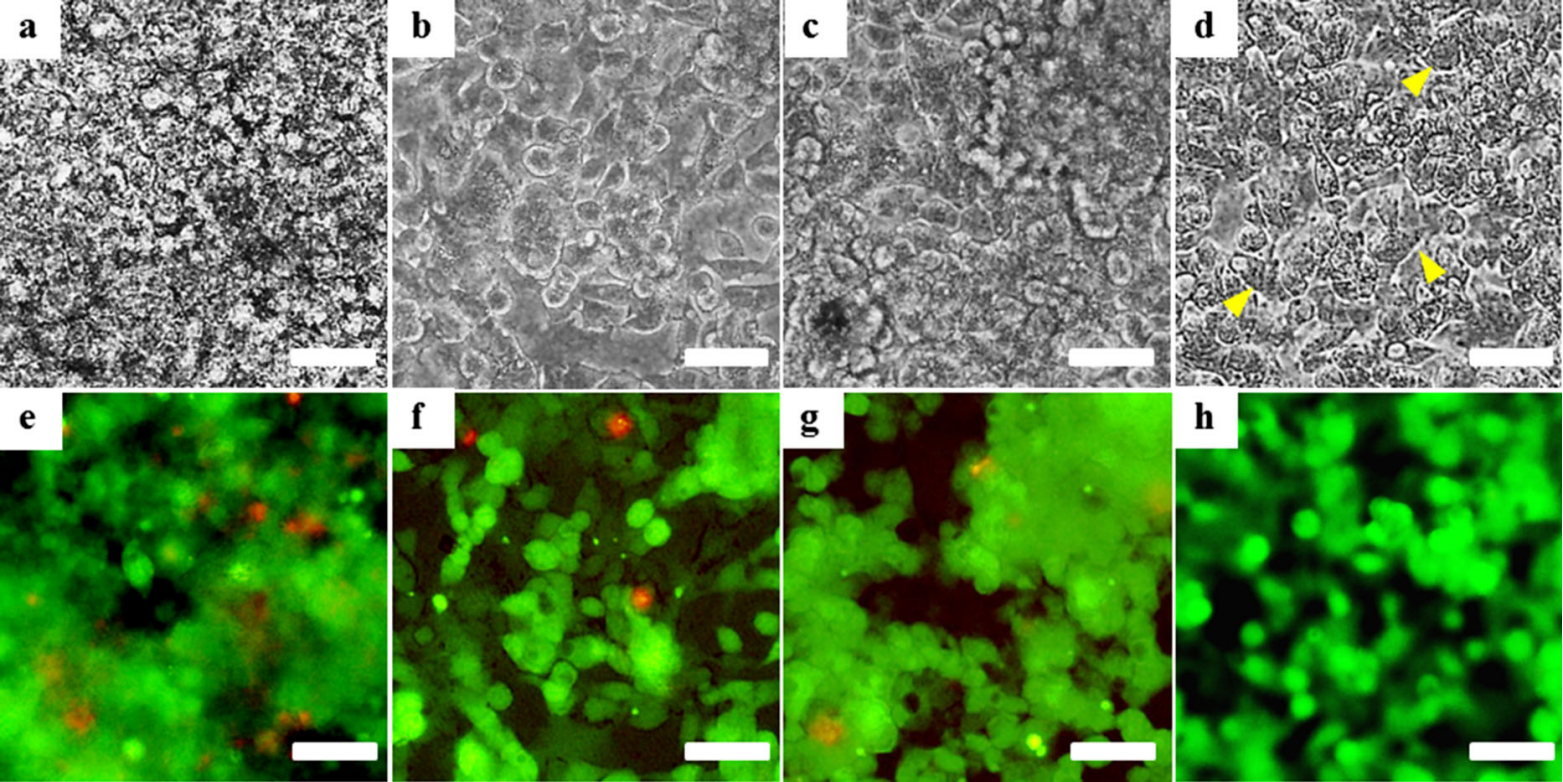
Microscopic observation of HepG2 cells cultured in control Millicell and CVM chambers for 3 days. HepG2 cells seeded in a control Millicell chamber with a 1.0 µm-porous PET membrane (**a**, **e**) were cultured on the liquid–liquid interface. Also, the cells seeded in CVM chambers pre-set on the solid surface were cultured on the liquid–solid interface for 48 h to induce the uniform cell growth and subsequently were cultured for 24 h on the liquid–solid (**b**, **f**), liquid–liquid (**c**, **g**) and liquid–gas interfaces (**d**, **h**). The same visual field of each culture system was observed with a phase-contrast microscope (**a**–**d**) and a fluorescent microscope (**e**–**h**) after staining the cells with calcein (*green*) and ethidium homodimer-1 (*red*). Arrowheads indicate typical bile canaliculus-like aspects (**d**). *Scale bars* represent 50 µm. (Color figure online)

HepG2 cells seeded in control Millicell and CVM chambers attached well to both 1.0 µm-porous PET membrane and CVM within 8 h. On day 1, the cells cultured on the liquid–solid interface using CVM chambers spread well and most of individual cells represented cobblestone or rectangle in shape although those on the liquid–liquid interface using control Millicell chambers formed many islands composed of relatively small cells shaped in round (data not shown). After that, the cells in both chambers proliferated to the stage of almost confluence until day 3 (Fig. 3a, d). Here, the cells cultured on the liquid–solid and liquid–gas interfaces using CVM chambers revealed epithelial cell-specific morphology (Fig. 3b, d) although those on the liquid–liquid interfaces using control Millicell and CVM chambers showed the tendency to form multicellular aggregates (Fig. 3a, c). Especially, the cells cultured on the liquid–gas interface reproduced the hepatic cord-like pattern involving typical bile canaliculus-like aspects (Fig. 3d). Also, the cells cultured on the liquid–gas interface were almost alive and healthy although those on the liquid–solid and liquid–liquid interfaces using CVM chambers as well as the liquid–liquid interface using control Millicell chambers were partially dead (Fig. 3e–h).

### Hepatic functions of HepG2 cells

**Fig. 4:**
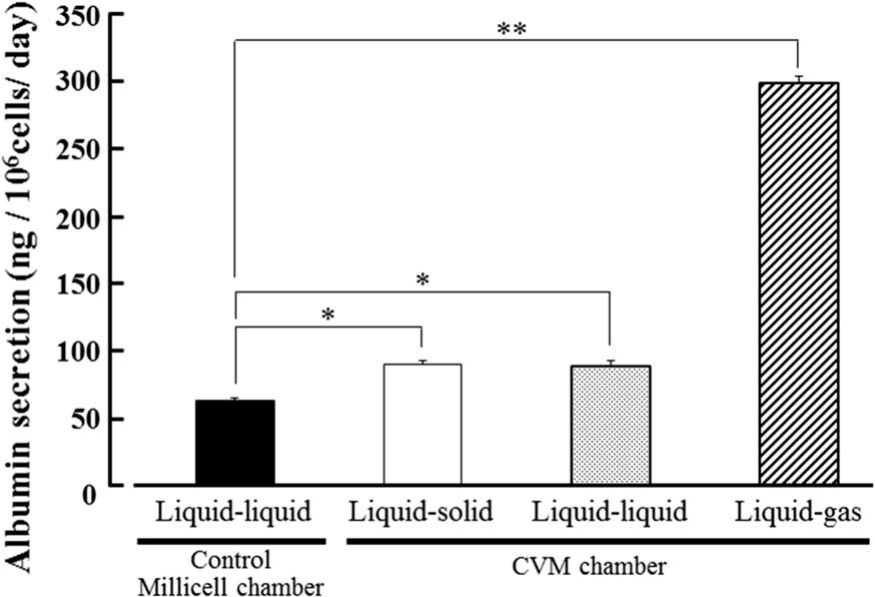
Albumin secretion level of HepG2 cells at day 3 in each culture method. *Closed*, *open*, *dotted*, and *hatched columns* represent the liquid–liquid interface culture method using a control Millicell chamber with a 1.0 µm-porous PET membrane, the liquid–solid, liquid–liquid and liquid–gas interface culture methods using CVM chambers, respectively. Each value represents the mean ± SD (n = 3). *P < 0.01 and **P < 0.001

**Fig. 5:**
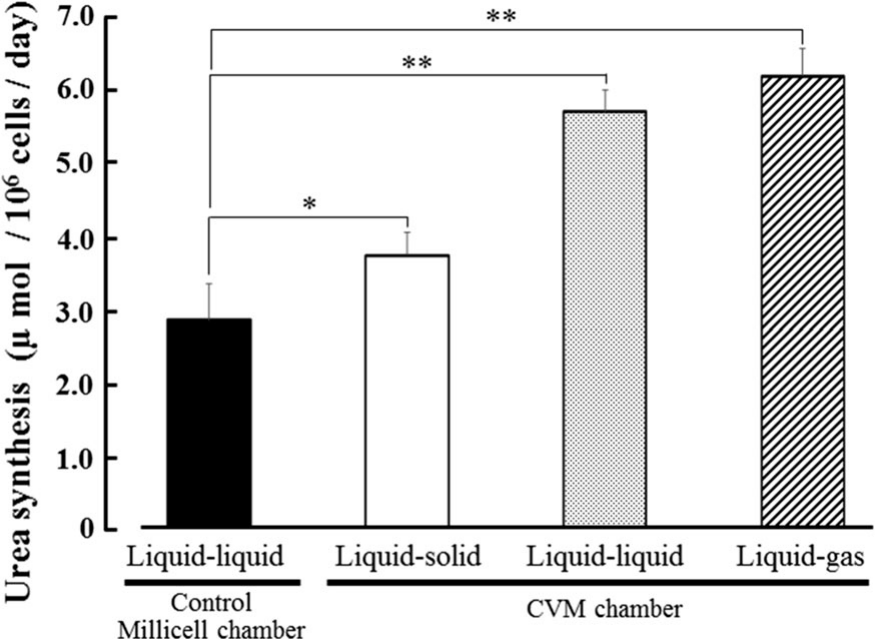
Urea synthesis level of HepG2 cells at day 3 in each culture method. *Closed, open, dotted, and hatched* *columns* represent the liquid–liquid interface culture method using a control Millicell chamber with a 1.0 µm-porous PET membrane, the liquid–solid, liquid–liquid and liquid–gas interface culture methods using CVM chambers, respectively. Each value represents the mean ± SD (n = 3). *P < 0.01 and **P < 0.001

**Fig. 6:**
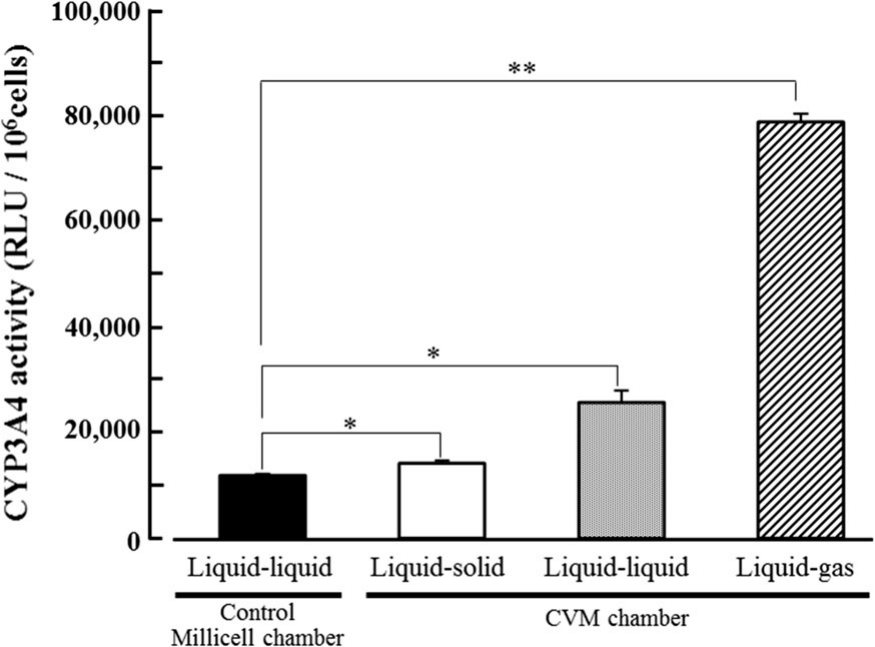
CYP3A4 activity level of HepG2 cells at day 3 in each culture method. *Closed*, *open*, *dotted*, and *hatched columns* represent the liquid–liquid interface culture method using a control Millicell chamber with a 1.0 µm-porous PET membrane, the liquid–solid, liquid–liquid and liquid–gas interface culture methods using CVM chambers, respectively. Each value represents the mean ± SD (n = 3). *P < 0.01 and **P < 0.001

Albumin secretion, urea synthesis and CYP3A4 activity levels of HepG2 cells at day 3 in each culture method were analyzed. As the results, albumin secretion level of the cells cultured on the liquid–solid, liquid–liquid and liquid–gas interfaces using CVM chambers was significantly higher than that in control culture and was 1.3, 1.3 and 4.7 times of control, respectively (Fig. 4). Also, urea synthesis level of the cells cultured on the liquid–solid, liquid–liquid and liquid–gas interfaces using CVM chambers was significantly higher than that in control culture and was 1.3, 2.0 and 2.2 times of control, respectively (Fig. 5). Similarly, CYP3A4 activity level of the cells cultured on the liquid–solid, liquid–liquid and liquid–gas interfaces using CVM chambers was significantly higher than that in control culture and was 1.2, 2.2 and 6.6 times of control, respectively (Fig. 6).

### ADME behavior of FD in HepG2 cells

**Fig. 7:**
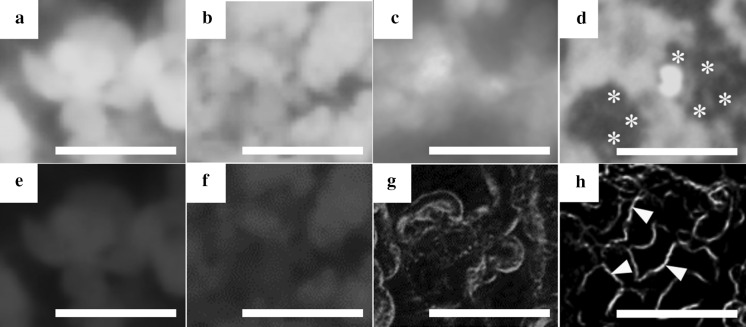
Fluorescence microscopic observation of FD-incorporated HepG2 cells at day 3 in each culture method. The cells were cultured on the liquid–liquid interface using a control Millicell chamber with a 1.0 µm-porous PET membrane (**a**, **e**), the liquid–solid (**b**, **f**), liquid–liquid (**c**, **g**) and liquid–gas (**d**, **h**) interfaces using CVM chambers. To assay the ADME of FD, the metabolized fluorescein at 10 min (**a**–**d**) and 60 min (**e**–**h**) after incorporating FD into the cells was observed under the same visual field in each culture system. *Asterisks* indicate the cells expressing the fluorescence in the interstices between the neighboring cells rather than inside the cells (**d**). *Arrowheads* indicate the excreted fluorescein into the bile canaliculus-like networks (**h**). *Scale bars* represent 50 µm

HepG2 cells at 10 min after incorporating FD showed a uniform and abundant fluorescent expression in the cells in each culture method (Fig. 7a–d). These data suggest that the cells after absorbing FD uniformly distributed it in cytosol and subsequently metabolized it into fluorescein quickly. Moreover, some cells in the liquid–gas interface culture method expressed the fluorescence in the interstices between the neighboring cells rather than inside the cells (Fig. 7d), suggesting that such cells had already started to excrete the fluorescein into the extracellular pockets. The fluorescence expression gradually decreased in the cells in each culture method as the time increased. The cells at 60 min after incorporating FD revealed the distinct fluorescence expression in the interstices between the neighboring cells in the liquid–gas interface culture method (Fig. 7h), however they represented the faint fluorescent expression around the multicellular aggregates in the liquid–liquid interface culture method (Fig. 7g) and in the cells in control and the liquid–solid interface culture methods (Fig. 7e, f). These data suggest that the cells in the liquid–gas interface culture method constructed two excretion pathways of fluorescent into the bile canaliculus-like networks and the extracellular solution, HBSS. However, the cells in the control, the liquid–solid and liquid–liquid interface culture methods seemed to merely utilize an inherent excretion pathway of fluorescence into the extracellular solution because they could not form such bile canaliculus-like networks.

## Discussion

The focus of this study is to develop a novel culture method for activating the liver-specific functions of HepG2 cells and to investigate its utility for analyzing the ADME behavior of a model chemical, FD. The reasons are firstly that HepG2 cells are considered to be excellent not only for extrapolating human due to human cells without species difference but also for maintaining reproducibility in cell culture experiments due to a cell strain compared as primary parenchymal hepatocytes and hepatocytes artificially differentiated from stem cells such as iPS (induced pluripotent stem) cells, ES (embryonic stem) cells and MSCs (mesenchymal stem cells) (Bouma et al. 1989; Knowles et al. 1980; Katenz et al. 2007; Kajiwara et al. 2012; Agarwal et al. 2008; Yin et al. 2015). Secondly, traditional culture methods for activating the liver-specific functions of HepG2 cells have crucial disadvantages that they required complicated culture techniques for a long period of more than 10 days (Evenou et al. 2010; Kikuchi et al. 2009; Ohono et al. 2008). Thirdly, FD was utilized for merely confirming the formation of bile canaliculi in most of the previous studies (Bi et al. 2006; Sudo et al. 2004; Swift et al. 2010).

In this study, we proposed a novel culture method with continuous oxygenation towards HepG2 cells using a CVM chamber (Fig. 1c) and confirmed that the CVM chamber set on the gas surface could preserve the culture medium poured inside (Fig. 2c). This phenomenon was also confirmed at 37 °C in a humidified atmosphere of 5 % CO_2_ and 95 % air (data not shown), suggesting that the culture medium-immersed CVM was equivalent to the atmosphere. In other words, the culture medium in the CVM chamber set on the gas surface can directly incorporate oxygen from both its upper and bottom surfaces (Fig. 1c) whereas that on the solid and liquid surfaces can not do so (Fig. 1a, b). Also, we confirmed that the control Millicell chambers set on the solid and gas surfaces leaked the culture medium (Fig. 2d, f, g, i). From the above findings, we designed three different culture methods on the liquid–solid, liquid–liquid and liquid–gas interfaces for CVM chambers and one control culture method on the liquid–liquid interface for control Millicell chambers. Here, the prior culture on the liquid–solid interface for 2 days was essential for obtaining HepG2 cells uniformly adhered to the CVM of CVM chambers on the liquid–liquid and liquid–gas interfaces because the CVM slightly deflected (data not shown). On day 3, at 24 h after the prior culture for 2 days, HepG2 cells cultured on the liquid–gas interface using CVM chambers revealed high viability in comparison to those cultured in the other conditions and formed the hepatic cord-like pattern involving typical bile canaliculus-like aspects never observed in the other culture methods (Fig. 3). It is reported that primary parenchymal hepatocytes required much oxygenation in tissue assemblies in vitro (Anada et al. 2012; Rotem et al. 1994; Schwartlander et al. 2007). Therefore, the above findings are considered that HepG2 cells were rapidly activated by the oxygenation from beneath the CVM and consequently they could grow well in maintaining excellent viability and showed the differentiated morphology of parenchymal hepatocytes. Also, the albumin production and urea synthesis levels are calculated as 263–315 ng/10^6^ cells/day and 6.1–7.9 µmol/10^6^ cells/day from the experiments using freshly isolated human hepatocytes as previously reported, respectively (Lu et al. 2011; Katenz et al. 2007). Therefore, it is considered that the liver-specific functions of HepG2 cells cultured on the liquid–gas interface using CVM chambers were activated to the almost same level of freshly isolated human hepatocytes in the albumin secretion (Fig. 4) and urea synthesis levels (Fig. 5). Thus, we developed a novel culture method for rapidly activating the liver-specific functions and morphology of HepG2 cells possessing excellent viability by combining the first liquid–solid interface culture for 48 h and the following liquid–gas interface culture for 24 h using CVM chambers.

Next, we examined the metabolic enzyme activity of CYP3A4 using a Luciferin-IPA substrate and calculated its relative luminescence unit (RLU) per 10^6^ cells. Consequently, the CYP3A4 activity of HepG2 cells cultured on the liquid–gas interface using CVM chambers and on the liquid–liquid interface using control Millicell chambers at day 3 was 79,000 ± 1300 RLU/10^6^cells and 11,900 ± 283 RLU/10^6^cells, respectively (Fig. 6). Meanwhile, the activity of HepG2 cells cultured on the conventional plastic plate at day 3 was 1010 ± 311 RLU/10^6^cells (data not shown). On the other hand, it is generally considered that freshly isolated human hepatocytes possess the wide range of CYP3A4 activities because their characteristics are dependent upon liver of donors. From this viewpoint, HepaRG cells are of current interest. Because, HepaRG cells that are a newly developed human hepatoma cell line and can differentiate into hepatocyte-like morphology by the treatment with DMSO are known to be useful for the studies on drug metabolism in vitro (Kanebratt and Andersson 2008). Regarding the CYP3A4 activity, it was reported that differentiated HepaRG cells cultured for 7 days on the conventional plastic plate represented about 160,000 RLU/10^6^ cells in the same assay as used in our current study (Mueller et al. 2014). Here, if we assume that the data without the appropriate standard curve can be compared, the CYP3A4 activity of HepG2 cells cultured on the conventional plastic plate, on the liquid–liquid interface using control Millicell chambers and on the liquid–gas interface using CVM chambers was about 1/160, 3/40 and 1/2 levels of differentiated HepaRG cells, respectively. In other words, even HepG2 cells can express the CYP3A4 activity considered to be utilized in near future for drug metabolism studies in vitro by culturing them on the liquid–gas interface using CVM chambers.

Further, we investigated the ADME behavior of FD by observing HepG2 cells at 10 and 60 min after incorporating it for 60 min and subsequently washing the cells. It was found that HepG2 cells rapidly absorbed and metabolized the distributed FD in cytosol into fluorescein in all culture conditions, and subsequently the cells cultured on the liquid–gas interface rapidly excreted it into both the bile canaliculus-like networks and the extracellular solution although the cells cultured in other conditions relatively slowly excreted it merely into the extracellular solution (Fig. 7). These findings suggest that HepG2 cells cultured on the liquid–gas interface express not only MRP3 and MRP4 for renal elimination but also MRP2 for fecal elimination as efflux transporters although the cells cultured in other conditions do the formers only (Godoy et al. 2013; Wojtal et al. 2006). Therefore, we concluded that HepG2 cells cultured on the liquid–gas interface using CVM chambers was useful for contentiously analyzing the ADME behavior of chemicals.

This is a first report describing that the liver-specific functions of HepG2 cells were easily activated by the two-step culture method using a CVM chamber; the first culture on the liquid–solid interface for 2 days and the following culture on the liquid–gas interface for 1 day. Here, the culture method was appropriate for not only maintaining cell viability but also inducing hepatic cord-like morphology. Also, it was demonstrated that the 3 days-culture model was useful for analyzing the ADME behavior of a model chemical, FD. Hereafter, we intend to fabricate a highly functional liver organoid utilizing the current model. As a next step, we are planning to incorporate the co-culture system using non-parenchymal cells (Bhatia et al. 1999; Corlu et al. 1997; Evenou et al. 2010; Kikuchi et al. 2009; Nishida and Taniguchi 2013; Ohono et al. 2008) and/or the sandwich culture system using traditional collagen gel (Bi et al. 2006; Sudo et al. 2004; Swift et al. 2010) into the present culture method.

## Abbreviations

ADME: Absorption, distribution, metabolism, and excretion
CYP: Cytochrome P450
CVM: Collagen vitrigel membrane
CXM: Collagen xerogel membrane
PET: Polyethylene terephthalate
FD: Fluorescein diacetate
DMEM: Dulbecco’s modified Eagle medium
HEPES: *N*-2-Hydroxyethylpiperazine-*N*′-2-ethanesulfonic acid
FBS: Fetal bovine serum
HBSS: Hanks’ balanced salt solution
DMSO: Dimethyl sulfoxide
PBS: Phosphate buffered saline
RLU: Relative luminescence unit
MRP: Multidrug resistance-associated proteins
